# Supplementary material for: A reinforcement learning model to inform optimal decision paths for HIV elimination
Source: Math Biosci Eng. Author manuscript; Available in PMC 2021 Nov 25. (PMC8613448; doi:10.3934/mbe.2021380)
Supplement: Appendix [file NIHMS1741109-supplement-Appendix.docx]

**A reinforcement learning model to inform optimal decision paths for HIV elimination**

**Seyedeh N. Khatami and Chaitra Gopalappa***

Mechanical and Industrial Engineering Department, University of Massachusetts Amherst, Amherst, MA 01003, USA

*** Correspondence:** Email: chaitrag@umass.edu; Tel: +1-4135452306.

**Appendix**

1. **Depicting MDP as a decision tree**

The sequential decision-making problem for HIV epidemic control and elimination can be visualized as a decision-tree. Figure A1 presents the decision tree for a simple 2-state system with $n$ action choices, with the epidemic state in 2015 being the start node, actions being the decision nodes, epidemic states at 5-year intervals being the chance nodes, the possible epidemic states in 2070 being the end nodes, and a policy being a path in a decision-tree. The problem is to identify, from among all possible paths of a decision-tree, the one with the maximum total reward. The decision tree starts in the initial state $S_{0}$ and corresponds to the epidemic state in the year 2015 ${(t}_{0}).$ Each square is a decision node at which there are multiple actions (stems branching out from a node) to choose from ($[a_{1},a_{2},\ldots a_{n}]$ in Figure A1). An action taken in any year $t_{i}$ transitions the epidemic to a different state in year $t_{i+5}$, with some uncertainty (oval) in the actual state it transitions to, denoted as probabilities $p_{a}(j,k)$ for transitioning to state $S_{k}$ from state $S_{j}$ when action $a$ is taken. Thus, the decision tree branches out from the year 2015 to 2070, with decision-making occurring at every 5-year interval. The “total reward” is the output at the end of each branch in the year 2070. However, the problem cannot be solved using decision trees because it is impractical to evaluate all possible paths exhaustively. In this simple 2-state $n$-actions example, that would be $2n$ paths if evaluating up to time $t_{1}$, $\left( 2n \right)^{2n}$ paths if evaluating up to time $t_{2}$, thus growing exponentially over the time-periods evaluated.

The HIV model introduced in this paper is significantly more extensive and has 16,501-states 36-actions evaluated for 11 time-periods. Hence, we formulated the problem as a Markov decision process (MDP), solved using optimization modeling that efficiently uses mathematical concepts to search through the solution space. MDP can also be solved by dynamic programming or reformulating as a linear programming optimization model; however, these methods also require the knowledge of the transition probability matrices ($P_{a}$) for each action $a$, square matrices with dimensions equal to the number of states ($p_{a}(j,k)$ are elements of $P_{a}$), which is infeasible for HIV (Gosavi, Simulation-based optimization: Parametric optimization techniques and reinforcement learning, 2003). Therefore, to overcome these challenges, we use Q-learning with a stochastic dynamic simulation model to directly simulate the state transitions (replacing $P_{a}$) and estimate rewards.


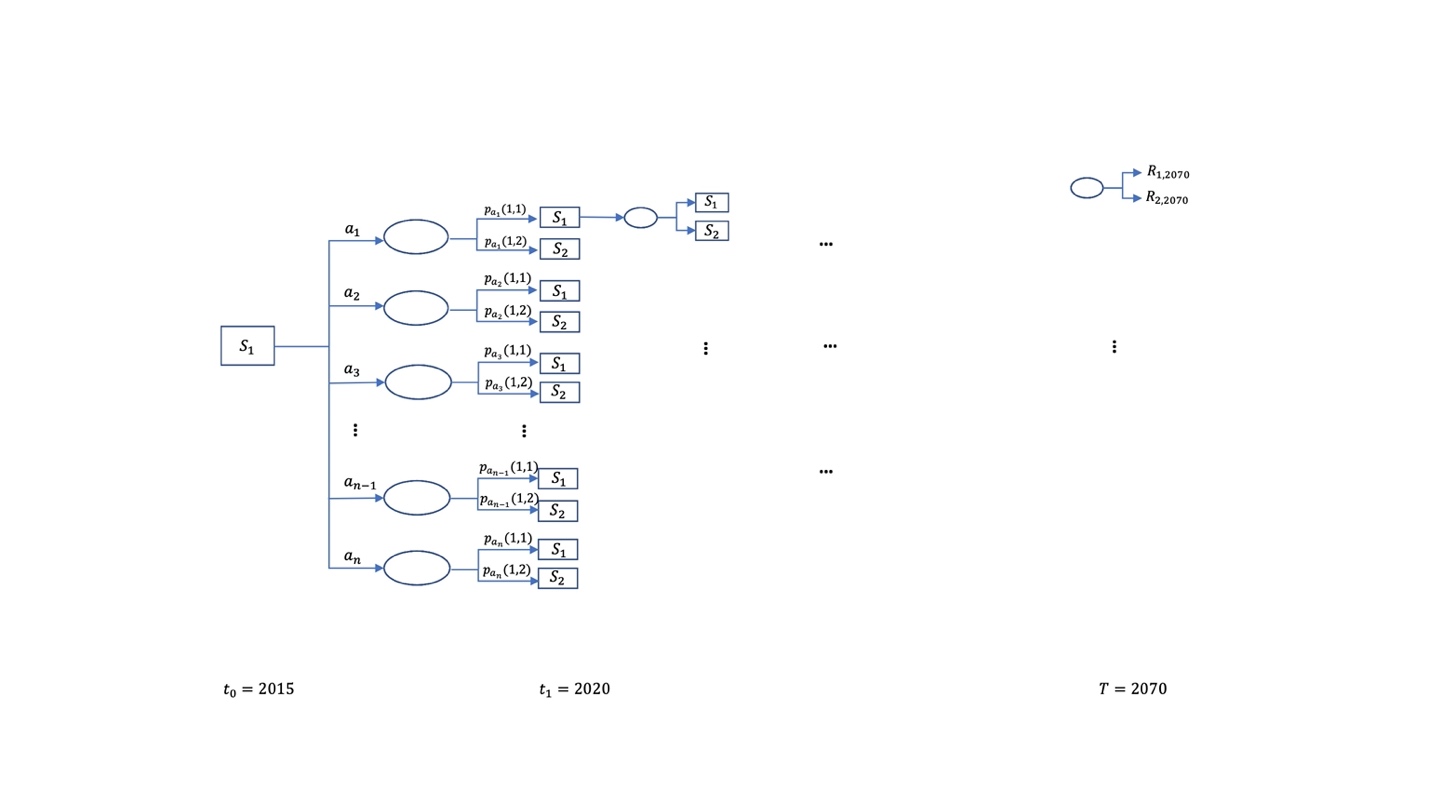


**Figure A1**. Decision tree representation of the Markov decision process problem (MDP); the objective of MDP is to find the path with an optimal trade-off in costs and benefits.

1. **Estimating diagnostic and retention-in-care rates for a given action and simulating the action in PATH 2.0 model**

As discussed in the main manuscript, we formulated an action as a combination of the percentage decrement in proportion unaware and the percentage increment in proportion on ART; each varying by transmission risk-group ($a_{unaware,i},a_{ART,i}, \forall i\in\left\{ HETs,MSM \right\}$). Corresponding to every action, we estimated the diagnostic rate ($\delta_{t})$ and retention-in-care rate (${1-\rho}_{t})$, and used the rates for simulating the action in the Progression and Transmission of HIV (PATH 2.0) model [2], including estimating the number of persons successfully intervened (newly diagnosed and retained-in-care) and the corresponding intervention costs. The estimation method includes a combination of expressing the system as a differential equations model and utilizing the PATH 2.0 model. In this section, we present the method of estimating $\delta_{t}$ and ${1-\rho}_{t}$ using $a_{unaware,i}$ and $a_{ART,i}$, the corresponding number of persons successfully intervened (newly diagnosed and retained-in-care), and simulating the action in PATH 2.0. In addition, we present the estimation of the intervention costs of that action in Appendix Section 4.

We can express the disease incidence and transition along the stages of care-continuum of HIV-infected persons as a set of differential equations or a compartmental model, with each disease and care continuum stage representing a compartment (Figure A2). We have four compartments, susceptible (S), infected and unaware (U), infected and aware but not in care (NC), and infected in care and on ART (ART).


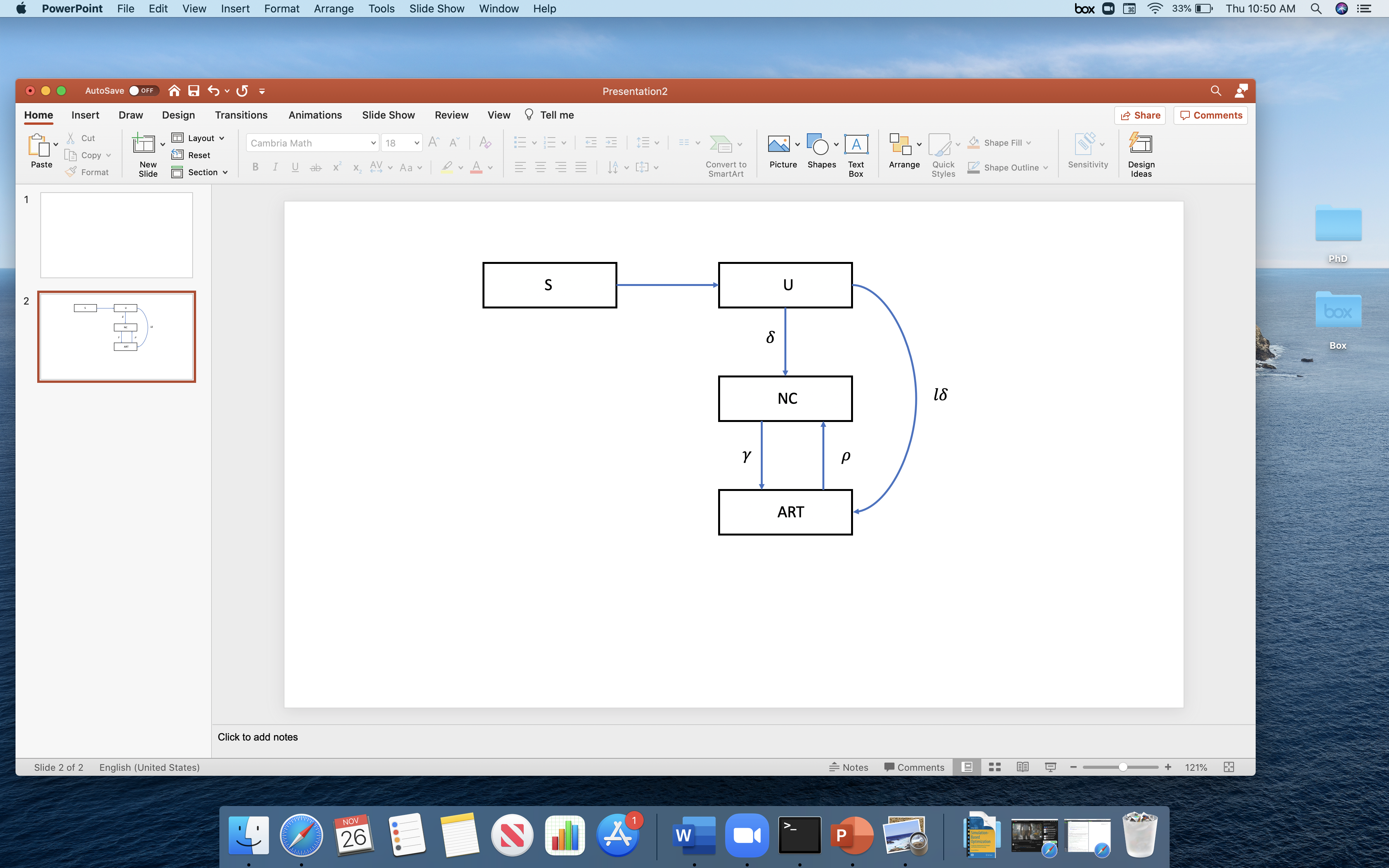


**Figure A2.** Flow diagram for disease incidence and transitioning along the stages of care-continuum HIV infected persons. S: susceptible, U: infected and unaware, NC: infected and aware but not in care, ART: infected, in care and on ART, $\delta$: diagnostic rate, $\gamma$: rate of entering care and treatment among those not in care, and ρ: rate of dropping-out of care, and $l\delta$: rate of diagnosis and linked to care.

Let,

$I_{t}$ be the number of infected persons at time $t$,

$i_{t}$ be the number of new infections at time $t$,

$p_{t,s}$ be the proportion of infected persons in stage $s \in\{U, NC, ART\}$, such that $p_{t,s}I_{t}$, would be the number of infected people in stage $s$ at time $t$,

$\delta_{t}$ be the diagnostic rate at time $t$,

$l_{t}$ be the proportion of persons linking to care and initiating treatment upon diagnosis, among those diagnosed at time $t$ ,

$\gamma_{t}$ be the rate of entering care and treatment among those not in care at time $t$,

$\rho_{t}$ be the rate of dropping-out of care and treatment at time $t$, and

$m_{t}$ be the number of new deaths at time $t$.

Then, at a sufficiently small incremental time-step $t+1$ (we use monthly increments), we can write the equations for the number of people in each stage by formulating as a system of differential equations,

| $p_{t+1,s}I_{t+1}=p_{t,s}I_{t}+\frac{dp_{t,s}I_{t}}{dt}$ | (1) |
| --- | --- |

where, $\frac{dp_{t,s}I_{t}}{dt}$ is the rate of the change in $p_{t,s}I_{t}$, i.e., the change in the number of infected persons in stage $s$ at $t$.

Specifically, for each stage $s \in\{U,NC,ART\}$ we can apply Eq (1) and write,

| $I_{t}p_{t,U}=I_{t-1}p_{t-1,U}+i_{t}-\delta_{t}I_{t-1}p_{t-1,U}$ | (2) |
| --- | --- |

| $I_{t}p_{t,NC}=I_{t-1}p_{t-1,NC}+\delta_{t}\left( 1-l_{t} \right)I_{t-1}p_{t-1,U}-\gamma_{t}I_{t-1}p_{t-1,NC}+\rho_{t}I_{t-1}p_{t-1,ART}$ | (3) |
| --- | --- |

| $I_{t}p_{t,ART}=I_{t-1}p_{t-1,ART}+\delta_{t}l_{t}I_{t-1}p_{t-1,U}+\gamma_{t}I_{t-1}p_{t-1,NC}-\rho_{t}I_{t-1}p_{t-1,ART}$ | (4) |
| --- | --- |

Additionally, as $p_{t,s}$ is the ‘proportion’ of infected people in stage $s$, summing over all disease stages should add to 1, or

| $p_{t,NC}=1-\left( p_{t,U}+p_{t,ART} \right)$ | (5) |
| --- | --- |

Further, the number of infected people at $t$ ($I_{t}$) would be the number infected at $t-1$ plus new infections ($i_{t}$) minus deaths ($m_{t}$), i.e.,

| $I_{t}=I_{t-1}+i_{t}-m_{t}$ | (6) |
| --- | --- |

Note, these equations are applied for each risk group (heterosexuals, and MSM) separately, but we do not indicate the risk group in subscripts for clarity of notations.

**Estimate diagnostic rate ($\boldsymbol{\delta}_{\mathbf{t}}$) and the corresponding number of persons successfully intervened (newly diagnosed)**

We can write the monthly change in proportion unaware as $-\left( p_{t-1,U}-p_{t,U} \right)$*=* $\hat{a}_{unaware,t}$, which is the selected proxy action choice for the change in proportion unaware at decision epoch T divided by 60 months $(i.e., \hat{a}_{unaware,t}\in\frac{a_{unaware,i}}{60})$, as our decision-making interval is every five years. Note that $\hat{a}_{unaware,t}$ is specific to risk-group $i$ but we drop subscript $i$ for clarity of notation. We calculate monthly estimates as the time-step in the PATH 2.0 simulation model is monthly. We assume the changes in proportion unaware are achieved uniformly over the 60-month period, and thus, the estimated diagnostic rate would be representative of linearly scaling up HIV-testing interventions over this interval. Then, using Eq (6), we can express diagnostic rate by rewriting Eq (2) as

| $\delta_{t}=\frac{-\left( I_{t-1}+i_{t}-m_{t} \right)p_{t,U}+I_{t-1}p_{t-1,U}+i_{t}}{I_{t-1}p_{t-1,U}} .$ | (7) |
| --- | --- |

By substituting $p_{t,U}=p_{t-1,U}+\hat{a}_{unaware,t}$ in Eq (7) we can write

| $\delta_{t}=\frac{-\left( I_{t-1}+i_{t}-m_{t} \right)\left( p_{t-1,U}+\hat{a}_{t,unaware} \right)+I_{t-1}p_{t-1,U}+i_{t}}{I_{t-1}p_{t-1,U}}$ | (8) |
| --- | --- |

and the corresponding number of persons to diagnose as

| $\delta_{t}I_{t-1}p_{t-1,U}=-\left( I_{t-1}+i_{t}-m_{t} \right)\left( p_{t-1,U}+\hat{a}_{t,unaware} \right)+I_{t-1}p_{t-1,U}+i_{t}.$ | (9) |
| --- | --- |

**Estimate retention-in-care rate and the number of persons successfully intervened (retained-in-care)**

We can write the monthly change in proportion on ART as $\left( p_{t,ART}-p_{t-1,ART} \right)=\hat{a}_{ART,t}$, which is the selected proxy action choice for the change in proportion on ART at decision epoch T divided by 60 months $(i.e., \hat{a}_{ART,t}\in\frac{a_{ART,i}}{60})$, as our decision-making interval is every five years. Note that, $\hat{a}_{ART,t}$ is specific to risk-group $i$, but we drop subscript $i$ for clarity of notation. We assume the changes in proportion on ART are achieved uniformly over the 60 month period, and thus, the estimated retention-in-care rates would be representative of linearly scaling up retention-in-care interventions over this interval. We set $p_{t,ART}=p_{t-1,ART}+\hat{a}_{ART,t}$ in Eq (4) and rewrite it to express the rate of dropping-out as

$$I_{t}p_{t,ART}=I_{t-1}p_{t-1,ART}+\delta_{t}l_{t}I_{t-1}p_{t-1,U}+\gamma_{t}I_{t-1}p_{t-1,NC}-\rho_{t}I_{t-1}p_{t-1,ART},$$

$$\rho_{t}=\frac{-\left( I_{t-1}+i_{t}-m_{t} \right)p_{t,ART}+I_{t-1}p_{t-1,ART}+\delta_{t}l_{t}I_{t-1}p_{t-1,U}+\gamma_{t}I_{t-1}p_{t-1,NC}}{I_{t-1}p_{t-1,C}},$$

| $\rho_{t}=\frac{-\left( I_{t-1}+i_{t}-m_{t} \right)\left( p_{t-1,ART}+\hat{a}_{ART,t} \right)+I_{t-1}p_{t-1,ART}+\delta_{t}l_{t}I_{t-1}p_{t-1,U}+\gamma_{t}I_{t-1}p_{t-1,NC}}{I_{t-1}p_{t-1,ART}},$ | (10) |
| --- | --- |

and estimate the corresponding number of persons dropping-out of care as

| $\rho_{t}I_{t-1}p_{t-1,ART}=-\left( I_{t-1}+i_{t}-m_{t} \right)\left( p_{t-1,ART}+\hat{a}_{ART,t} \right)+I_{t-1}p_{t-1,ART}+\delta_{t}l_{t}I_{t-1}p_{t-1,U}+\gamma_{t}I_{t-1}p_{t-1,NC}.$ | (11) |
| --- | --- |

Following from above, we estimate the number of persons retained in care as the number of persons not dropping-out of care, i.e., $(1-\rho_{t})I_{t-1}p_{t-1,ART}$.

**Simulating the action in PATH 2.0**

Simulating any given action $a_{unaware,i},a_{ART,i}, \forall i\in\left\{ HETs,MSM \right\}$ over the 5-year interval (in monthly time-steps) involves iteratively simulating PATH every month for estimating the terms on the right-hand side of Eqs (8) and (10), solving for diagnostic rate ($\delta_{t}$) and retention-in-care rate $(1-\rho_{t})$ for that month using (8) and (10), estimating the numbers to diagnose and retain-in-care using Eqs (9) and (11), and using that in PATH to simulate diagnosis and care events for that month. Details of the PATH 2.0 model along with the estimation of the parameters in Eqs (8) and (10) and simulating care and diagnosis events are discussed elsewhere [2], here we only give a brief description related to the estimation of parameters in Eqs (8) and (10). PATH 2.0 model was initialized to be representative of the HIV infected population in the US in 2006, using data from the US National HIV Surveillance System (NHSS), and simulated to 2015. It keeps track of $I_{t}$ and $p_{t,s}$ over time. It estimates new infections ($i_{t}$) by modeling transmissions at the individual-level for every susceptible-infected partnership, modeling it as a function of stage $s \in\{U, NC, ART\}$ of the infected person at time $t-1$. Thus, the number of new infections is a function of $p_{t-1,U}$ and $p_{t-1,NC}$. It estimates the number of deaths ($m_{t}$) by simulating mortality at the individual-level for each person using stage- and age-specific mortality rates. It uses annual data from NHSS for the proportion linking to care ($l_{t}$) upon diagnosis and assume it will be maintained at the 2015 level for future years [3]. It uses re-entry rates ($\gamma_{t}$) from studies in the literature [4].

# **Mathematical advantage of formulating action space as changes in proportions unaware and on ART instead of diagnostic and retention-in-care rates**

As discussed in section 2, we formulate the action space ($A$) as a combination of changes in proportions unaware and on ART, i.e., $A=\left\{ \left[ a_{unaware,i},a_{ART,i}, \forall i\in\left\{ HETs,MSM \right\} \right] \right\}$, as proxy for diagnostic and retention-in-care rates $\left\{ \left[ \delta_{i},1-\rho_{i}, \forall i\in\left\{ HETs, MSM \right\} \right] \right\}$. We prove here that the proxy metrics efficiently constrain the size of the action space, which would increase the chance of convergence of the Q-learning algorithm. It is also computationally efficient as it requires fewer evaluations of the simulation model. We also prove that formulating an action as $\left[ \Delta\delta_{i},1-\Delta\rho_{i}, \forall i\in\left\{ HETs, MSM \right\} \right]$ also leads to a large action space, where $\Delta\delta_{i}$ and $1-\Delta\rho_{i}$ are changes in $\delta_{i}$ and $\rho_{i}$, respectively, over two consecutive decision epochs, as the proxies $a_{unaware,i}$ and $a_{ART,i}$ are also decrements or increments (of $\mu_{u,i}$ or $\mu_{v,i}$, respectively) over two consecutive decision epochs. We also prove that, corresponding to every combination of $\left[ \hat{a}_{unaware,t},\hat{a}_{ART,t} \right]$ there is a unique combination of $\left[ \delta_{t},{1-\rho}_{t} \right]$, and thus solving for the optimal combination of the proxy metrics is equivalent to solving for the optimal diagnostic and retention-in-care rates. We prove these through Remarks 1 to 4.

Note that, $\left[ \hat{a}_{unaware,t},\hat{a}_{ART,t} \right]$ and $\left[ \delta_{t},{1-\rho}_{t} \right]$ have $t$ (time) subscripts as they are the monthly values defined in Appendix Section 2 corresponding to a proxy action $\left[ a_{unaware,i},a_{ART,i}, \forall i \right]$ and original action $\left\{ \left[ \delta_{i},1-\rho_{i}, \forall i \right] \right\}$, respectively. They do vary by risk-group but we drop the subscripts $i$ for clarity of notations. Without loss of generality, we use $\left[ \hat{a}_{unaware,t},\hat{a}_{ART,t} \right]$ and $\left[ \delta_{t},{1-\rho}_{t} \right]$ to prove our Remarks about $\left[ a_{unaware,i},a_{ART,i}, \forall i\in\left\{ HETs,MSM \right\} \right]$ and $\left[ \delta_{i},1-\rho_{i}, \forall i\in\left\{ HETs, MSM \right\} \right]$, respectively.

**Remark 1**: Given the system state $x$ at time $t-1$, ($X_{t-1}=x$), corresponding to every action, $a_{unaware,i}$, there is a unique diagnostic rate, $\delta_{t}$, i.e., $f:\hat{a}_{unaware,t} \to\delta_{t}$ is a bijection function and corresponding to every action, $a_{ART,i}$, there is a unique retention-in-care rate$,{1-\rho}_{t}$, i.e., $g:\hat{a}_{ART,t} \to1-\rho_{t}$ is a bijection function.

**Proof**: From Eq (8) we have diagnostic rate as:

the only controllable unknown is $\hat{a}_{unaware,t}$ as all other parameters correspond to or are calculated using system state at time $t-1$, as discussed in Section 2. Therefore, $\delta_{t}$ is a linear function of $\hat{a}_{unaware,t}$ , i.e., $f:\hat{a}_{unaware,t} \to\delta_{t}$ is a bijection function. This implies that, at any given system state at time $t-1$ for every action $\hat{a}_{unaware,t}$, we can calculate a unique value for the diagnostic rate.

Similarly, from Eq (10) we have drop-out rate as:

the only controllable unknown is $\hat{a}_{ART,t}$ as all other parameters correspond to or are calculated using system state at time $t-1$, as discussed earlier in Section 2. Therefore, $\rho_{t}$ is a linear function of $\hat{a}_{ART,t}$, i.e., $g:\hat{a}_{ART,t}\to\rho_{t}$ is a bijection function. This implies that, at any given system state at time $t-1$, for every action $a_{ART}$, we can calculate a unique value for the retention-in-care rate ${1-\rho}_{t}$.

**Remark 2**: From a public health perspective, all actions that result in $\left( p_{t-1,U}-p_{t,U} \right)<0$ or $\left( p_{t,ART}-p_{t-1,ART} \right)<0$ are undesirable and should not be selected.

**Proof**: If the conditions are true, it would imply that a larger proportion of people with HIV are unaware of their infection and/or are not on treatment at time t compared to $t-1$. Being unaware and not on treatment are associated with an increase in transmissions and mortalities. Thus, all actions that result in $\left( p_{t-1,U}-p_{t,U} \right)<0$ or $\left( p_{t,ART}-p_{t-1,ART} \right)<0$ are undesirable as they worsen the epidemic and should not be selected.

**Remark 3**: Setting action space as $A=\{\left[ a_{unaware,i},a_{ART,i},\forall i \right]\}$ instead of $A=\{\left[ \delta_{i},1-\rho_{i},\forall i \right]\}$ efficiently controls the number of possible interventions and thus is more computationally efficient.

**Proof:** If action space $A=\left\{ \left[ a_{\mathrm{unaware}},a_{ART} \right] \right\}:$

As $\hat{a}_{unaware,t}=-\left( p_{t-1,U}-p_{t,U} \right)$, we can directly select actions such that $-\left( p_{t-1,U}-p_{t,U} \right)\geq$0 (see Remark 2). As we use $a_{\mathrm{unaware}}$ to estimate diagnostic rate, it constrains diagnostic rates to only those that correspond to desirable outcomes. Thus, all testing intervention programs that are below the minimum diagnostic rates can be excluded. The selection of action is not dependent on any parameters of the system, except for the feasibility constraint $p_{t,U}>10\%,$ as discussed in Section 2.1 of the manuscript.

Similarly, as $a_{ART}=-\left( p_{t-1,ART}-p_{t,ART} \right)$, we can directly select actions such that $\left( p_{t,ART}-p_{t-1,ART} \right)\geq$0 (see Remark 2), naturally constraining the selection of retention-in-care rates to those that would result in desirable outcomes. Thus, all intervention programs whose efficacy is below the minimum retention-in-care rates estimated here can be excluded. The selection of action is not dependent on any parameters of the system, except for the feasibility constraint $p_{t,ART}<90\%,$ as discussed in the Section 2.1.

Rearranging Eq (7) for diagnostic rate from above, we can write

| $\delta_{t}=\frac{-\left( i_{t}-m_{t} \right)p_{t,U}+I_{t-1}\left( p_{t-1,U}-p_{t,U} \right)+i_{t}}{I_{t-1}p_{t-1,U}}$ | (12) |
| --- | --- |

And further rearranging to write

| $\left( p_{t-1,U}-p_{t,U} \right)=\frac{\delta_{t}I_{t-1}p_{t-1,U}-\left[ i_{t}-\left( i_{t}-m_{t} \right)p_{t,U} \right]}{I_{t-1}}$ | (13) |
| --- | --- |

As $p_{t,U}\leq1$, if $i_{t}>m_{t}$ then $-\left( i_{t}-m_{t} \right)p_{t,U}<i_{t}$; and if $i_{t}<m_{t}$ then $-\left( i_{t}-m_{t} \right)p_{t,U}>0$. Therefore, the following condition is always true $[i_{t}-\left( i_{t}-m_{t} \right)p_{t,U}]>0$.

The above implies that, there are certain values of $\delta_{t}$ such that $\delta_{t}I_{t-1}p_{t-1,U}\geq\left[ i_{t}-(i_{t}-m_{t})p_{t,U} \right]$, which would yield $\left( p_{t-1,U}-p_{t,U} \right)\geq0$, and certain other values of $\delta_{t}$ such that $\delta_{t}I_{t-1}p_{t-1,U}<\left[ i_{t}-(i_{t}-m_{t})p_{t,U} \right]$, which would yield $\left( p_{t-1,U}-p_{t,U} \right)<0$, which is an undesirable outcome from a public health perspective. As the values of $\delta_{t}$ that generate $\delta_{t}I_{t-1}p_{t-1,U}\geq\left[ i_{t}-(i_{t}-m_{t})p_{t,U} \right]$ is time-dependent on values of $I_{t-1}, p_{t-1,U}, i_{t}, and m_{t}$, a large set of values for $\delta_{t}$ should be evaluated as part of the action space. This is computationally expensive, and moreover inefficient, as many cases would result in an undesirable outcome.

Rearranging the equation for drop-out rate from Eq (10), we can write

| $\rho_{t}=\frac{-\left( I_{t-1}+i_{t}-m_{t} \right)p_{t,ART}+I_{t-1}p_{t-1,ART}+\delta_{t}l_{t}I_{t-1}p_{t-1,U}+\gamma_{t}I_{t-1}p_{t-1,NC}}{I_{t-1}p_{t-1,ART}}$ | (14) | |
| --- | --- | --- |
| $\rho_{t}=\frac{-\left( i_{t}-m_{t} \right)p_{t,ART}-I_{t-1}{{(p}_{t,ART}-p}_{t-1,ART})+\delta_{t}l_{t}I_{t-1}p_{t-1,U}+\gamma_{t}I_{t-1}p_{t-1,NC}}{I_{t-1}p_{t-1,ART}}$ | | (15) | |
| ${{(p}_{t,ART}-p}_{t-1,ART})=\frac{-I_{t-1}\rho_{t}p_{t-1,ART}-\left( i_{t}-m_{t} \right)p_{t,ART}+\delta_{t}l_{t}I_{t-1}p_{t-1,U}+\gamma_{t}I_{t-1}}{I_{t-1}}$ | (16) | |

If $i_{t}>m_{t}$ then $\left( i_{t}-m_{t} \right)p_{t,ART}>0$ and as $+\delta_{t}l_{t}I_{t-1}p_{t-1,U}+\gamma_{t}I_{t-1}>0$ and $-I_{t-1}\rho_{t}p_{t-1, ART}<0$, there are certain combinations that can result ${{(p}_{t,ART}-p}_{t-1,ART})$ <0.

Similarly, if $i_{t}<m_{t}$ then $\left( i_{t}-m_{t} \right)p_{t,ART}<0$ and as $+\delta_{t}l_{t}I_{t-1}p_{t-1,U}+\gamma_{t}I_{t-1}>0$ and $-I_{t-1}\rho_{t}p_{t-1,ART}<0$, there are certain combinations that can result in ${{(p}_{t,ART}-p}_{t-1,ART})$ <0.

As the values of $\rho_{t}$ that result in ${{(p}_{t,ART}-p}_{t-1,ART})$ <0 are time-dependent on values of $I_{t-1}, p_{t-1,ART}, i_{t},$ and $m_{t}$ a large set of values for $\rho_{t}$ and thus $1-\rho_{t}$ should be part of the action space, which is again computationally expensive and inefficient as many cases would result in undesirable outcomes.

**Remark 4**: Setting action space as $A=\{\left[ a_{unaware,i},a_{ART,i},\forall i \right]\}$ instead of $A=\{\left[ {\Delta\delta}_{i},1-{\Delta\rho}_{i},\forall i \right]\}$ efficiently controls the number of possible interventions and thus is more computationally efficient.

**Proof**: If action space $A=\left\{ \left[ a_{unaware},a_{ART} \right] \right\}:$ We discuss this case in Remark 3.

If action space $A=\{\left[ {\Delta\delta}_{i},1-{\Delta\rho}_{i},\forall i \right]\}$ the corresponding rates to simulate at every time-step would be $\delta_{t}-\delta_{t+1}$ and $\rho_{t}-\rho_{t+1}$:

Without loss of generality, we prove this Remark by showing that to generate one scenario equivalent of $\left( p_{t-1,U}-p_{t,U} \right)=\left( p_{t,U}-p_{t+1,U} \right)=0$, i.e., keeping proportion unaware constant over two consecutive time-steps, it would require evaluations of multiple combinations of $\delta_{t}$ and $\delta_{t+1}$ as the combination that generates $\left( p_{t-1,U}-p_{t,U} \right)=\left( p_{t,U}-p_{t+1,U} \right)=0$ would be dependent on system parameters at that time.

Writing equations for $\delta_{t}$ and $\delta_{t+1}$, setting $\left( p_{t-1,U}-p_{t,U} \right)=\left( p_{t,U}-p_{t+1,U} \right)=0$, and subtracting one from the other, we get

| $\delta_{t}=\frac{-\left( i_{t}-m_{t} \right)p_{t,U}+i_{t}}{I_{t-1}p_{t-1,U}}$ | (17) |
| --- | --- |

| $\delta_{t+1}=\frac{-\left( i_{t+1}-m_{t+1} \right)p_{t+1,U}+i_{t+1}}{I_{t}p_{t,U}}$ | (18) |
| --- | --- |

Subtracting Eqs (17) and (18) we get:

| $\delta_{t}-\delta_{t+1}=\frac{-\left( i_{t}-m_{t} \right)p_{t,U}+i_{t}}{I_{t-1}p_{t-1,U}}- \frac{-\left( i_{t+1}-m_{t+1} \right)p_{t+1,U}+i_{t+1}}{I_{t}p_{t,U}}$ | (19) |
| --- | --- |

As $\left( p_{t-1,U}-p_{t,U} \right)=0$ and $\left( p_{t,U}-p_{t+1,U} \right)=0$, we can set $p_{t-1,U}=p_{t,U}=p_{t+1,U}$:

| $\delta_{t}-\delta_{t+1}=\frac{-\left( i_{t}-m_{t} \right)p_{t,U}+i_{t}}{I_{t-1}p_{t,U}}- \frac{-\left( i_{t+1}-m_{t+1} \right)p_{t,U}+i_{t+1}}{I_{t}p_{t,U}}$ | (20) |
| --- | --- |

| $\delta_{t}-\delta_{t+1}= \frac{I_{t}m_{t}p_{t,U}+I_{t}i_{t}\left( 1-p_{t,U} \right)-I_{t-1}m_{t+1}p_{t,U}-I_{t-1}i_{t+1}\left( 1-p_{t,U} \right)}{I_{t}I_{t-1}p_{t,U}}$ | (21) |
| --- | --- |

| $\delta_{t}-\delta_{t+1}= \frac{\left( I_{t}m_{t}-I_{t-1}m_{t+1} \right)p_{t,U}+\left( I_{t}i_{t} -I_{t-1}i_{t+1} \right)\left( 1-p_{t,U} \right)}{I_{t}I_{t-1}p_{t,U}}.$ | (22) |
| --- | --- |

Therefore,$\left( p_{t-1,U}-p_{t,U} \right)=\left( p_{t,U}-p_{t+1,U} \right)=0$ could result from a range of diagnostic rate changes, $\delta_{t}-\delta_{t+1}>0,$ $<0,$ or $=0$ depending on values of $I_{t},m_{t},I_{t-1},m_{t+1}, i_{t},and i_{t+1}$. This implies that, if formulating an action as $\left[ {\Delta\delta}_{i},1-{\Delta\rho}_{i},\forall i \right]$, a large subset of values for $\delta_{t}-\delta_{t+1}$ should be evaluated as part of the action space. This is computationally expensive and, moreover, inefficient as many cases would result in an undesirable outcome.

Similarly, without loss of generality, to generate one scenario equivalent of $\left( p_{t-1,ART}-p_{t,ART} \right)=\left( p_{t,ART}-p_{t+1,ART} \right)=0$, i.e., keeping proportion on ART constant over two consecutive time-steps, it would require evaluations of multiple combinations of $\rho_{t} \mathrm{and}\rho_{t+1}$ as the combination that generates $\left( p_{t-1,ART}-p_{t,ART} \right)=\left( p_{t,ART}-p_{t+1,ART} \right)=0$ would be dependent on system parameters at that time.

Writing equations for $\rho_{t}$ and $\rho_{t+1}$, setting $\left( p_{t-1,ART}-p_{t,ART} \right)=\left( p_{t,ART}-p_{t+1,ART} \right)=0$, and subtracting one from the other, we get

| $\rho_{t}=\frac{-\left( i_{t}-m_{t} \right)p_{t,ART}+\delta_{t}l_{t}I_{t-1}p_{t-1,U}+\gamma_{t}I_{t-1}p_{t-1,NC}}{I_{t-1}p_{t-1,ART}}$ | (23) |
| --- | --- |

| $\rho_{t+1}=\frac{-\left( i_{t+1}-m_{t+1} \right)p_{t+1,V}+\delta_{t+1}l_{t+1}I_{t}p_{t,U}+\gamma_{t+1}I_{t}p_{t,NC}}{I_{t}p_{t,ART}}$ | (24) |
| --- | --- |

| $\rho_{t}-\rho_{t+1}=\frac{-\left( i_{t}-m_{t} \right)p_{t,ART}+\delta_{t}l_{t}I_{t-1}p_{t-1,U}+\gamma_{t}I_{t-1}p_{t-1,NC}}{I_{t-1}p_{t-1,ART}}-\frac{-\left( i_{t+1}-m_{t+1} \right)p_{t+1,ART}+\delta_{t+1}l_{t+1}I_{t}p_{t,U}+\gamma_{t+1}I_{t}p_{t,NC}}{I_{t}p_{t,ART}}$ | (25) |
| --- | --- |

As $\left( p_{t-1,ART}-p_{t,ART} \right)=0$ and $\left( p_{t,ART}-p_{t+1,ART} \right)=0$, we can set

$p_{t-1,ART}=p_{t,ART}=p_{t+1,ART}$*:*

| $\rho_{t}-\rho_{t+1}=\frac{-\left( i_{t}-m_{t} \right)p_{t,ART}+\delta_{t}l_{t}I_{t-1}p_{t-1,U}+\gamma_{t}I_{t-1}p_{t-1,NC}}{I_{t-1}p_{t,ART}}-\frac{-\left( i_{t+1}-m_{t+1} \right)p_{t,ART}+\delta_{t+1}l_{t+1}I_{t}p_{t,U}+\gamma_{t+1}I_{t}p_{t,NC}}{I_{t}p_{t,ART}}$ | (26) |
| --- | --- |

Rewriting with a common denominator,

| $\rho_{t}-\rho_{t+1}=\frac{\left[ \begin{aligned} I_{t}m_{t}p_{t,ART}-I_{t}i_{t}p_{t,ART}+I_{t-1}i_{t+1}p_{t,ART}-I_{t-1}m_{t+1}p_{t,ART} \\ + I_{t}\left( \delta_{t}l_{t}I_{t-1}p_{t-1,U}+\gamma_{t}I_{t-1}p_{t-1,NC} \right)-I_{t-1}\left( \delta_{t+1}l_{t+1}I_{t}p_{t,U}+\gamma_{t+1}I_{t}p_{t,NC} \right) \end{aligned} \right]}{I_{t}I_{t-1}p_{t,ART}}$ | (27) |
| --- | --- |

Rearranging the numerator,

| $\rho_{t}-\rho_{t+1}=\frac{\left[ \begin{aligned} {[I}_{t}{(m}_{t}-i_{t})+I_{t-1}{(i_{t+1}-m}_{t+1})]p_{t,ART} \\ +I_{t}\left( \delta_{t}l_{t}I_{t-1}p_{t-1,U}+\gamma_{t}I_{t-1}p_{t-1,NC} \right)-I_{t-1}(\delta_{t+1}l_{t+1}I_{t}p_{t,U}+\gamma_{t+1}I_{t}p_{t,NC}) \end{aligned} \right]}{I_{t}I_{t-1}p_{t,ART}}$ | (28) |
| --- | --- |

Therefore,$\left( p_{t-1,ART}-p_{t,ART} \right)=\left( p_{t,ART}-p_{t+1,ART} \right)=0$ could result from a range of drop-out rate changes, $\rho_{t}-\rho_{t+1}>0$*,* $<0,$ or $=0$, depending on values of $I_{t},m_{t},I_{t-1},m_{t+1}, i_{t},\mathrm{and}i_{t+1}$. This implies, if formulation an action as $\left[ {\Delta\delta}_{i},1-{\Delta\rho}_{i},\forall i \right]$, a large subset of values for $\rho_{t}-\rho_{t+1}$ should be evaluated as part of the action space. This is computationally expensive, and moreover inefficient, as most of the values would result in an undesirable outcome.

From Remarks 3 and 4, we can conclude that formulating the action space as $A=\left\{ \left[ \delta_{i},1-\rho_{i}, \forall i\in\left\{ HETs, MSM \right\} \right] \right\}$ would require evaluations of a large set of actions. For instance, if we use changes in testing and retention-in-care rates, we should consider both increase and decrease in these rates as both combinations can lead to higher proportion aware and proportion on ART. In this approach, choices would be combination of -25, 0, and 25% for testing rate and -20, -10, 0, 10, and 20% for retention-in-care (3 x 5 for HETs x 3 x 5 for MSM = 225). Or if we use changes in testing frequency and changes in retention-in-care rate as the action space, choices could be to test every 1, 2, 3, …, 10 years and retention-in-care rate can change by -20, -10, 0, 10, and 20% (10 x 5 for HETs x 10 x 5 for MSM = 2500). Problems with large action space generate issues of convergence, becoming infeasible to solve, and moreover, in this case, computationally inefficient, as a considerable portion of those values would result in $\left( p_{t-1,U}-p_{t,U} \right)<0$, which is an undesirable outcome from a public health perspective (Remark 2). On the contrary, using action space $A=\left\{ \left[ a_{unaware,i},a_{ART,i}\forall i \right] \right\}$ would naturally constrain the action space (to size 36 as discussed in paper section 2.1) by removing those actions that would result in undesirable outcomes, and is thus more efficient. Further, Remark 1 concludes that, for any given system state, solving for the optimal action $\left[ a_{unaware,i},a_{ART,i}\forall i \right]$ is equivalent to solving for optimal $\left[ \delta_{i},1-\rho_{i}, \forall i\in\left\{ HETs, MSM \right\} \right]$.

# **Cost functions**

We estimated the total cost of an action as the summation of the corresponding cost of the testing intervention program, retention-in-care intervention program, and treatment. The treatment costs are estimated in the PATH 2.0 simulation model by applying regimen-specific costs at the individual-level and are discussed elsewhere [2]. In section 2, for every action, we estimated the number of persons successfully intervened (numbers newly diagnosed and retained-in-care). Corresponding to those numbers, we discuss the estimation of the corresponding costs of testing and retention-in-care intervention programs in this section.

## *Estimation of HIV testing costs*

In the estimation of testing costs, we make the following assumptions based on currently available data on testing behavior and testing intervention programs [4,5,6]. HIV testing programs can be conducted in clinical or non-clinical settings, each having its own fixed and variable costs [5, 6]. Fixed cost includes the cost of clinics, other infrastructure, devices, equipment, staff, etc., while variable cost includes the cost per person tested. The marginal variable cost per additional person tested is a non-linear function of the proportion of the population tested and is influenced by the type of outreach program needed. Some people get tested voluntarily and incur only the cost of testing, while some get tested as a result of implementing an outreach intervention and thus incur additional costs of intervention [5]. Outreach intervention can include providers reaching out to the client's community, delivering health information, reaching populations who have not been part of the traditional healthcare delivery system, HIV awareness campaigns, etc. [7]. The outreach intervention program is not 100% effective, meaning that not all outreached persons would get tested for HIV. To achieve the required number of persons tested under any action, outreach programs maybe be necessary. Under any given system state for proportion unaware, the corresponding cost of outreach is a non-linear function of the number of people outreached, i.e., the marginal cost to achieve one additional HIV-positive test increases as the proportion unaware decreases indicating more efforts would be needed to reach a larger portion of the population [8, 9]. The cost per person for persons testing positive is different from the cost per person for persons testing negative, as persons testing positive also undergo follow-up confirmation tests and additional care services for linkage to care [10, 11]. In accordance with current CDC recommendations, we assumed only persons with high risk are recommended for regular testing and applied testing costs for only these populations. We assumed that 6% of heterosexual females, 10% of heterosexual males, and all MSM are high-risk [12, 13, 14].

We estimate the cost of testing corresponding to an action $a$ as follows:

Let

$r_{t,a}$ be the number of persons testing positive at time step t under action $a$ ,

$n_{t,a}$ be the number of persons testing negative at time step t under action $a$,

$x_{t,a}$ be the number of persons reached through an outreach testing intervention program at time step t under action $a$,

$Y_{v}$ be the variable cost per person testing positive,

$X_{v}$ be the variable cost per person testing negative,

$O_{v}$ be the variable cost per person outreached through an outreach testing intervention program,

$X_{f-cl,a}$ be the total fixed cost of testing in a clinical setting under action $a$,

$X_{f-ncl,a}$ be the total fixed cost of testing in a non-clinical setting under action $a$,

$X_{f-o,a}$ be the total fixed cost of implementing an outreach intervention program under action $a$, and

$C_{a_{t}}$ be the total cost of testing under action $a$.

Then, we can calculate the total cost of testing action as

| $C_{a_{t}} = r_{t,a}Y_{v}+x_{t,a}O_{v}{+n}_{t,a}X_{v}+X_{f-cl,a}+X_{f-ncl,a} + X_{f-o,a}.$ | (29) |
| --- | --- |

Note that $r_{t,a}$ is the number of persons successfully intervened, i.e., the persons newly diagnosed as estimated in Appendix section 2. To achieve this, a larger number of persons must have tested negative ($n_{t,a}$), and further, a larger number of persons must have been reached through an intervention ($x_{t,a}$) as it is not necessary that all who are intervened would take a HIV test. Therefore, using our estimates of $r_{t,a}$ and based on other data from the literature on the effectiveness of interventions programs, we estimate $n_{t,a}$ and $x_{t,a}$. Furthermore, as the unit costs for persons in each category ($r_{t,a}$, $n_{t,a}$, or $x_{t,a}$) are also likely different, we estimate the unit costs for each category ($Y_{v}$, $O_{v}$, $X_{v}$, respectively) in addition to the fixed costs using data from the literature. Below, we discuss the estimation of each of these components on the right-hand side of the (29).

**Estimation of $\boldsymbol{r}_{\boldsymbol{t,a}}$-** Number of persons testing positive at time step t under action $a$:

Note that this is the number of persons successfully intervened, i.e., the persons newly diagnosed as estimated in Appendix section 2, i.e.,

| $r_{t,a}=\delta_{t}I_{t-1}p_{t-1,U}$ | (30) |
| --- | --- |

where

$\delta_{t}$ is the rate of HIV diagnosis under action $a,$

$I_{t}$ is the number of infected persons at time $t$, and

$p_{t,U}$ is the proportion of infected persons in stage unaware at time *t*.

Estimation of $\delta_{t}$ was discussed earlier, and $I_{t-1}$ and $p_{t-1,U}$ are simulated in the PATH 2.0 model.

**Estimation of** $\boldsymbol{x}_{\boldsymbol{t,a}}$ - Number of people outreached at time step t under action $a$:

Let

$\mu$ be the rate of diagnosis through voluntary testing,

$\theta$ be the effectiveness of an outreach intervention program,

$\varphi$ be the proportion of people testing positive for HIV among those tested through an outreach intervention program, and

$N_{t}$ be the total population at time *t*.

Then we can write,

$\mu I_{t-1}p_{t-1,U}$ as the number of persons diagnosed through voluntary testing, and

$x_{t,a}\theta\varphi$ as the number of persons diagnosed through an outreach testing intervention program,

i.e.,

| $x_{t,a}\theta\varphi={(\delta}_{t}I_{t-1}p_{t-1,U})-\left( \mu I_{t-1}p_{t-1,U} \right)$ | (31) |
| --- | --- |

And thus, estimate the total number of people outreached as

| $x_{t,a}=\frac{{(\delta}_{t}I_{t-1}p_{t-1,U})-({\mu I}_{t-1}p_{t-1,U})}{\theta\varphi}$ | (32) |
| --- | --- |

We calculate the unknown terms on the right-hand side of the above equation as follows,

$\mu=\frac{number of persons newly diagnosed with HIV in 2010}{I_{2010}p_{2010,U}}$ by assuming that the rate of HIV diagnosis in the year 2010, which is just prior to the implementation of the first HIV national strategic plan [15], is the rate of diagnosis through voluntary testing and that it remains the same for future years,

$\theta=30\%$ an assumption taken from [6], and

$\varphi=\left( \frac{I_{t-1}}{N_{t-1}} \right)\left( \varphi_{InterventionGroup,year}/\frac{I_{Detroit,year}}{N_{Detroit,year}} \right)$,

where $\varphi_{InterventionGroup,year}=0.0153$ is the proportion of persons testing positive in the outreach intervention program [5], and $\frac{I_{Detroit,year}}{N_{Detroit,year}}=0.0057$ is the prevalence of HIV in Detroit at the time the study was conducted[[1]](#footnote-1).

**Estimation of $\boldsymbol{n}_{\boldsymbol{t,a}}$-** Number of persons testing negative at time step t under action $a$:

Following from the previous subsection, we can write

$x_{t,a}\theta(1-\varphi)$ as the number of persons testing negative for HIV among those tested through an outreach intervention program, and

$\mu\left( N_{t}-I_{t-1}p_{t-1,U}-x_{t,a} \right)$ as the number of persons testing negative for HIV among those who voluntarily get tested.

Thus, we estimate the total number of HIV negative cases as:

| $n_{t,a}=\mu\left( N_{t}-I_{t-1}p_{t-1,U}-x_{t,a} \right)+x_{t,a}\theta\left( 1-\varphi\right)$ | (33) |
| --- | --- |

The estimation of all parameters on the right side of the equation has been discussed earlier.

**Estimation of $\boldsymbol{X}_{\boldsymbol{v}}$ -** Variable cost per person testing negative:

Let

$c_{r,n}$ be the rapid test cost for an HIV-negative case,

$c_{c,n}$ be the conventional test cost for an HIV-negative case,

$n_{n}$ be the notification cost for an HIV-negative case,

$c_{add}$ be the additional cost per person performed in a non-clinical setting,

$\tau$ be the proportion rapid test, and

$\alpha$ be the proportion tested in the clinical setting.

We can then estimate the variable cost per person for an HIV negative case as

| $X_{v}= \tau c_{r,n}+(1-\tau)c_{c,n}+n_{n}+\left( 1-\alpha\right)c_{add}$ | (34) |
| --- | --- |

For the parameters on the right-hand side of the equation, we use estimates from the literature [6] and are summarized in Table A1.

**Estimation of** $\boldsymbol{Y}_{\boldsymbol{v}}$ – Variable cost per person testing positive:

Let

$c_{r,p}$ be the rapid test cost for an HIV-positive case,

$c_{c,p}$ be the conventional test cost for an HIV-positive case,

$n_{r,p}$ be the rapid test notification cost for an HIV-positive case,

$n_{c,p}$ be the conventional test notification cost for an HIV-positive case, and

$c_{cnf}$ be the confirmatory cost.

Hence, we estimate the variable cost per person for a HIV positive case as follows:

| $Y_{v} =\tau\left( c_{r,p}+n_{r,p} \right)+(1-\tau)(c_{c,p}+ n_{c,p})+ c_{cnf}+(1-\alpha) c_{add}$ | (35) |
| --- | --- |

For the parameters on the right-hand side of the equation, we use estimates from the literature [6] and are summarized in Table A1.

**Estimation of** $\boldsymbol{O}_{\boldsymbol{v}}$- Variable cost per person outreached:

As the proportion of HIV-infected persons unaware of their infection decreases, the marginal cost of reaching one additional HIV-positive person increases. Therefore, we formulate the variable cost of outreach intervention as a non-linear function of increment in the proportion of people outreached relative to the number of people outreached in the base year with respect to the total population.

Let $O_{v_{0}}$be the base year variable cost for outreaching $x_{0}$ people among the total population in the base year $(2015)$, $x_{0}$ be the number of people outreached in the base year estimated using data in [5] with outreach intervention effectiveness of 30% [6], $\Delta x$ be the increment in the number of populations outreached from the base year with respect to total population at time t, calculated as $\frac{x_{t}-x_{0}}{N_{t}}$, and $w$ be the coefficient of a non-linear variable cost function.

Then per person variable cost of $\Delta x$ increment in outreaching $\mathrm{is}$:

| $O_{v}=O_{v_{0}}e^{\Delta xW}$ | (36) |
| --- | --- |

Derivation:

We assume that $\Delta x$ increase in the number of people outreached imposes an extra cost of $O_{v}w\Delta x$ and estimate per person outreach cost as,

$$O_{v+\Delta x}=O_{v}+O_{v}w\Delta x$$

$$O_{v+\Delta x}-O_{v}=O_{v}w\Delta x$$

$$\frac{O_{v+\Delta x}-O_{v}}{O_{v}}=w\Delta x$$

Integrating on both sides,

| $O_{v}=O_{v_{0}}e^{\Delta xW}.$ | (37) |
| --- | --- |

For the parameters on the right-hand side of the equation, we use estimates from the literature and are summarized in Table A2.

**Estimation of** $\boldsymbol{X}_{\boldsymbol{f-cl,a}}$– Total fixed cost of testing in a clinical setting corresponding to action $a$:

We assume that infected and uninfected persons share the same fixed costs in a clinical setting:

Let

$\alpha$ be the proportion of people testing in a clinical setting,

$m_{cl}$ be capacity of a clinic (we use an average estimate for clinic capacity),

$f_{c}$ be the total fixed cost per clinic with $m_{\mathrm{cl}}$ capacity, and

$X_{f-cl,a}$ be the total clinical fixed cost.

Then the total fixed cost for testing $\alpha{(r}_{t,a}+n_{t,a})$ number of people in a clinical setting is estimated as follows:

| $X_{f-cl,a}=\left\lceil\frac{{(r}_{t,a}+n_{t,a}) \alpha}{m_{cl}} \right\rceil f_{c}$ | (38) |
| --- | --- |

For the parameters on the right-hand side of the equation, which were not discussed earlier, we use estimates from the literature [11, 10, 5], and are summarized in Table A1.

**Table A1.** Cost Components of HIV Positive and Negative Testing[[2]](#footnote-2).

| Input | Value | Source |
| --- | --- | --- |
| Testing cost, including screen and confirmatory test |  |  |
| HIV-uninfected individual, rapid screen | $22.13 (2012$) | Based on cost components from [10, 11, 16]. |
| HIV-uninfected individual, conventional screen | Before 2016: $8.24 (2012$)  2016+: $10.36 (2012$) | Based on cost components from [11, 16, 17]. |
| HIV-infected individual, rapid screen | Before 2016: $86.70 (2012$)  2016+: $78.80 (2012$) | Based on cost components from [10, 11, 16, 17] and adjusted to 2012$. Assumes a repeat screen and a Western blot confirmatory test before 2016; a Geenius HIV 1/2 confirmatory screen is used in 2016 and after. |
| HIV-infected individual, conventional screen | Before 2016: $60.02 (2012$)  2016+: $58.91 (2012$) | Based on cost components from [11, 16, 17] and adjusted to 2012$. Assumed Western blot confirmatory test before 2016, and a Geenius HIV 1/2 confirmatory screen in 2016 and after. |
| NAT[[3]](#footnote-3), applied for discrepant Western blot confirmatory test | $160.07 (2012$) | [17] |
| Additional cost per test performed in non-clinical (vs. clinical) setting | $52.66 (2005$) | [5] |
| Notification costs |  |  |
| HIV-uninfected | $0.45 (2009$) | [16] |
| HIV-infected, conventional screen | $5.88 (2009$) | [16] |
| HIV-infected, rapid screen | $10.86 (2009$) | [16] |
| Outreach cost per test (when applied) | $13.67 (2005$) | [5] |

**Estimation of** $\boldsymbol{X}_{\boldsymbol{f-ncl,a}}$– Total fixed cost of testing in a non-clinical setting corresponding to action $a$:

We assume that infected and uninfected persons share the same fixed costs in non-clinical testing.

Let $m_{\mathrm{nc}}$ be the average capacity of a non-clinical setting and $f_{\mathrm{nc}}$ be the total fixed cost per non-clinic setting with capacity $m_{\mathrm{nc}}.$

Then total fixed cost to test ($1- \alpha$)($r_{t,a}+n_{t,a})$ people in a non-clinical setting is estimated as follow:

| $X_{f-ncl,a}=\left\lceil\frac{{(r}_{t,a}+n_{t,a}) (1-\alpha)}{m_{nc}} \right\rceil f_{nc}$ | (39) |
| --- | --- |

For the parameters on the right-hand side of the equation that were not discussed earlier, we use estimates from the literature [10, 11, 16, 17], and are summarized in Table A1.

**Estimation of** $\boldsymbol{X}_{\boldsymbol{f-o,a}}$- Fixed cost of outreach intervention:

Let

$m_{o}$ be the capacity of outreach intervention, and

$f_{o}$ be the total fixed cost of outreaching $x_{t}$ people.

Then the total fixed cost to outreach $x_{t,a}$ number of people is estimated as

| $X_{f-o,a}=\left\lceil\frac{x_{t}}{m_{o}} \right\rceil f_{o}$ | (40) |
| --- | --- |

For the parameters on the right-hand side of the equation that were not discussed earlier, we use estimates from the literature [5] and are summarized in Table A2.

**Table A2.** Summary of Parameters and Values Used in Testing and Outreach Intervention.

| Notation | Description | Value(s) | Reference |
| --- | --- | --- | --- |
|  | Base year variable cost for outreaching people | $16.59 (2015$) | [5] |
| $\Delta x$ | Increment in the number of populations outreached from the base year |  | Calculated in PATH |
| $W$ | coefficient of variable cost | 0.1, 0.2, 0.3 | Assumption |
| $m_{cl}$*,* $m_{nc}$ | Clinical/ non-clinical capacity | 1000 | Assumption |
|  | Total fixed cost per clinic with capacity | $56379 (2015$) | [5] |
|  | Total fixed cost per clinic with capacity | $64851 (2015$) | [5] |

**Estimation of retention-in-care cost**

We assume that the required proportion on ART under any action ($a)$ can be achieved by implementing retention-in-care programs that ensure patients remain in care and consistently take antiretroviral therapy treatment to achieve viral load suppression [18]. Retention-in-care programs could include a combination of a face-to-face meeting with patients at primary care visits, brief interim phone contacts between appointments, appointment reminders, and missed-visit calls [18].

We assume retention-in-care programs could include fixed and variable costs. Fixed costs include the cost of office space, durable items such as computers, printers, telephones, etc. And variable costs include staff time spent on management of patients and personal contact with patients [18]. We assume the variable cost per person is a non-linear function of the proportion of people retained in care, i.e., the marginal cost to achieve one additional person retained in care increases as the proportion on ART increases indicating more efforts would be needed to retain a larger number of people in care.

We estimate the cost of retention-in-care corresponding to an action as follows.

Let

$d_{t,a}$ be the number of persons retained in care at time step t under action $a$;

$R_{v}$ be the variable cost per person retained in care,

$E_{f,a}$ be the total fixed cost of retaining in care under action $a$, and

$C_{a_{r}}$ be the total cost of retention in care under action $a$.

Then, we can calculate the total cost retention in care action as:

| $C_{a_{r}} = d_{t,a}R_{v}+ E_{f,a}.$ | (41) |
| --- | --- |

Below we discuss the estimation of each component on the right-hand side of the equation.

**Estimation of $\boldsymbol{d}_{\boldsymbol{t,}a}$-** Number of people retained in care:

Note that, this is the number of persons successfully intervened (number retained-in-care) and estimated in Appendix Section 2, i.e.,

| $d_{t,a}=\left( 1-\rho_{t} \right)I_{t-1}p_{t-1,ART}$ | (42) |
| --- | --- |

The parameters on the right-hand side of the equation are calculated in the PATH 2.0 simulation model.

**Estimation of $\boldsymbol{R}_{\boldsymbol{v}}$-** Retention-in-care variable costs:

Let

$p_{{ART}_{0}}$ be the proportion on ART in the base year 2015, which is calculated in PATH,

$R_{{ART}_{0}}$be the base year variable cost of achieving $p_{\mathrm{ART}_{0}}$,

$\Delta p_{ART}$ is the increment in the proportion on ART with respect to the base year, and

$Y$ is the coefficient of the non-linear variable cost function.

Similar to testing outreach variable cost, we calculate the retention-in-care variable cost as follows:

| $R_{ART}=R_{{ART}_{0}}e^{\Delta p_{ART}Y}$ | (43) |
| --- | --- |

For the parameters on the right hand-side of the equation that were not discussed earlier, we use estimates from the literature [18] and are summarized in Table A3.

**Estimation of $\boldsymbol{E}_{\boldsymbol{f,a}}$-**Retention-in-care fixed costs:

Let

$m_{r}$ be the retention-in-care intervention setting capacity, and

$f_{r}$ be the fixed cost per retention-in-care intervention program (with capacity $m_{r}$).

We estimate the total fixed cost for retaining $d_{t,a}$ number of people in care as:

| $E_{f,a}=\left\lceil\frac{d_{t,a}}{m_{r}} \right\rceil f_{r}$ | (44) |
| --- | --- |

For the parameters on the right hand-side of the equation that were not discussed earlier, we use estimates from the literature [18] and are summarized in Table A3.

**Table A3.** Summary of Parameters Used in Retention-in-care Intervention.

| Notation | Description | Value(s) | Reference |
| --- | --- | --- | --- |
| $p_{{ART}_{0}}$ | Proportion on ART at time step 0 |  | Calculated in PATH |
|  | Base year variable cost of achieving | $117 (2015$)  $235 (2015$)  $300 (2015$) | Low, medium, and high retention-in-care costs [18] |
| $\Delta p_{ART}$ | Increment in the proportion on ART |  | Calculated in PATH |
| $Y$ | Coefficient of variable cost | 0.1, 0.2, 0.3 | Assumption |
| $m_{r}$ | Retention-in-care intervention program capacity | 500 | Assumption based on [18] |
|  | Fixed cost per retention-in-care intervention program with capacity | $17977 (2015$)  $22708 (2015$)  $29330 (2015$) | Low, medium, and high retention-in-care costs  [18] |


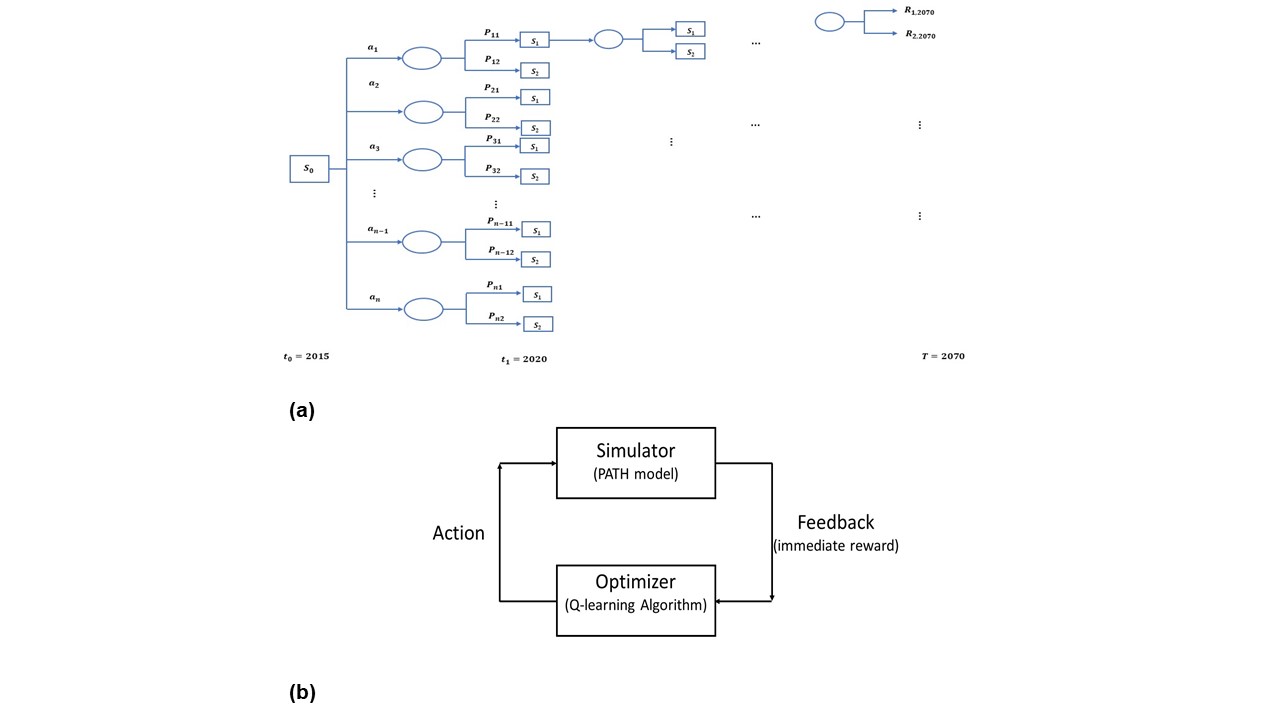


**Figure A3.** Schematic of the reinforcement learning (RL) algorithm. The RL algorithm takes action, and it is fed into the simulator. The simulator simulates the action and returns an immediate reward to the algorithm to update the following action accordingly.

# **Q-Learning algorithm**

**Table A4.** Finite-horizon Q-learning Algorithm to Identify Optimal Solutions to the Markov Decision Process [1].

| - Set $k =0$, $T=11$ (maximum number of decision stages) - Initialize Q-factor, $Q_{k} \left( x,t,a \right)$ to a very small negative number (i.e.${-10}^{-15}$) - While $k<k_{max}$ 1. Set $t=1$ and current state as $x$, setting PATH to start at HIV epidemic in the year 2015. 2. Select action, using decaying epsilon greedy [19], [20] $a\left( x,t \right)= \left\{ \begin{aligned} random selection from action space A with probability \epsilon_{t} \\ arg\max_{b\in A(x,t)} Q_{k}(x,t,b) with probability \left( 1-\epsilon_{t} \right) \end{aligned} \right.$ $\epsilon_{t}=\left\{ \begin{aligned} \frac{0.85}{k}+0.049 k\leq4000 \\ \frac{0.85}{k-3500}+0.049 k>4000 \end{aligned} \right.$ 3. Simulate the system using PATH, result in the transition state in $t+1$ to be . 4. Using the PATH model, calculate the immediate reward, $R_{a}\left( x,x^{'} \right).$ 5. Update $Q\left( x,t,a \right)$ as $Q_{k+1}\left( x,t,a \right)=\left( 1-\alpha\right)Q_{k}\left( x,t,a \right)+ \alpha\left[ R_{a}\left( x,x^{'} \right)+\gamma\max_{b\epsilon A\left( x^{'},t+1 \right)}Q_{k}\left( x^{'},t+1,b \right) \right]$  where,   - $\alpha$ is the step size a polynomial rate decaying with $k$ [21]. We assumed $\alpha= \frac{1}{{(k+1)}^{0.5625}}$ - $\gamma$ is the discount factor (We assume $\gamma$ =1).  1. Set $x$ ← $x^{'}$, and $t=t+ 1$ - if $t= T$ set $k=k+ 1$, and go to step 1 - else, go to step 2 - Identify optimal solution: optimal action $“a”$ to be taken at time $“t”$ when the system is in state   $x:$ $a\left( x,t \right)\epsilon arg\max_{b\epsilon A(x,t)} Q_{k_{max}} (x,t,b)$. |
| --- |

# **Testing convergence of the Q-learning reinforcement algorithm**

In this section, we present the uncertainty in results, specifically, the uncertainty in the optimal policy $\left[ a_{unaware,HET},a_{ART,HET},a_{unaware,MSM},a_{ART,MSM} \right]$ and the uncertainty in values of testing and retention-in-care rates $\left[ \delta,1-\rho\right]$, corresponding to each scenario for different values of Q-learning training iterations $k_{max}$ (2000, 3000, 4000, and 5000). An algorithm is said to have converged if, it has reached a local optima through the iterative search process, i.e., successfully solved for an optimal combination of testing and retention-in-care rates. If the number of iterations is not sufficiently large, there is a risk that the algorithm is terminated before convergence. The ideal number is typically determined through experimentation.

Further, there could be multiple local optima, i.e., multiple policies could yield similar total rewards, and because of the stochastic nature of the epidemic system, the optimal policy could be a range rather than a point estimate. Therefore, we ran the model for varying number of iterations, 2000, 3000, 4000, and 5000, and compared the corresponding total rewards, to ensure convergence and obtain the uncertainty range in optimal policies (Figures A4, A7, and A10 for each of the three cost functions). Note also that the training with $k_{max}=5000$ has an additional exploration after 4000 iterations (defined by the epsilon-greedy action selection defined in the main manuscript). The relative difference in the total population costs between the varying iterations were at most 2% in each cost function evaluated, suggesting convergence (Figures A6, A9, and A12 for each of the three cost functions). The changes in new infections and the number of people with HIV (PWH) were also minimal (Figures A5, A8, and A11 for each of the three cost functions). The corresponding optimal policies differed slightly, more so in future years than earlier years, suggesting stochastic uncertainty as the model projects further into the future (Figures Figure A4, A7, and A10 for each of the three cost functions). Therefore, in Results of the main paper, we present the range of optimal policies across these iterations as the uncertainty range.


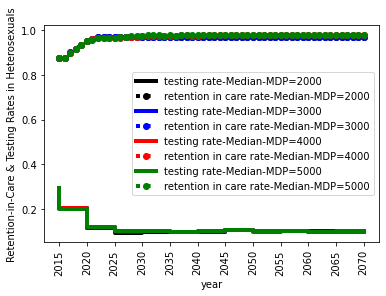

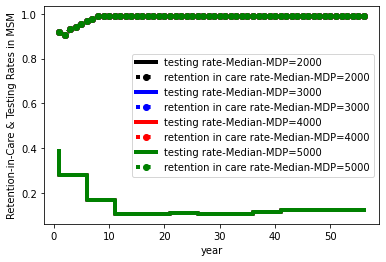


(a) (b)


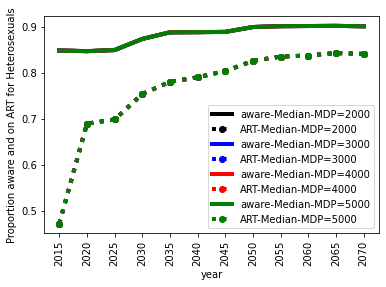

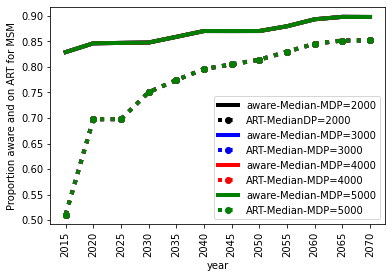


(c) (d)

**Figure A4.** Medium Cost Function: Combination of optimal policy (testing and retention-in-care rates) for heterosexuals (a) and MSM (b), and corresponding proportion of aware and on ART for heterosexuals (c) and MSM (d) for MDP iterations of 2000 (black), 3000 (blue), 4000 (red), and 5000 (green). Results are an average of 100 runs.


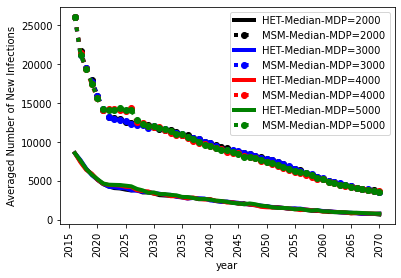

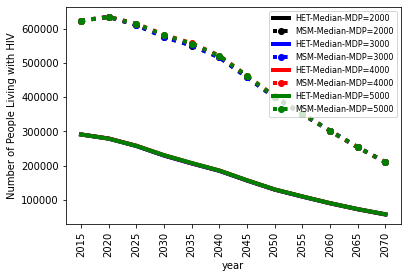


1. (b)

**Figure A5.** Median Cost Function: Impact of implementing a combination of optimal policy on the number of new infections (a) and the number of people living with HIV (b) for heterosexuals (solid lines) and MSM (dashed lines) for MDP iterations of 2000 (black), 3000 (blue), 4000 (red), and 5000 (green). Results are an average of 100 runs.


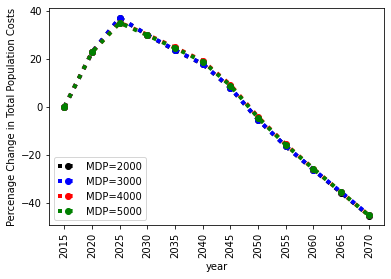


**Figure A6.** Median Cost Function: Percentage increment in total population cost of implementing optimal policy for MDP iterations of 2000 (black), 3000 (blue), 4000 (red), and 5000 (green). Results are an average of 100 runs.


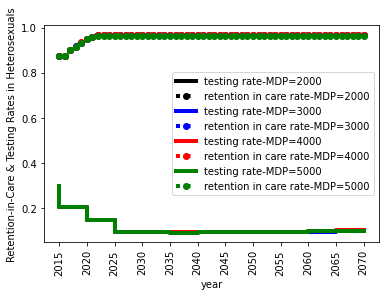

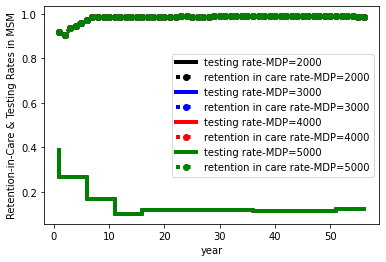


(a) (b)


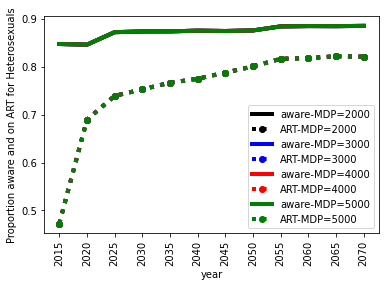

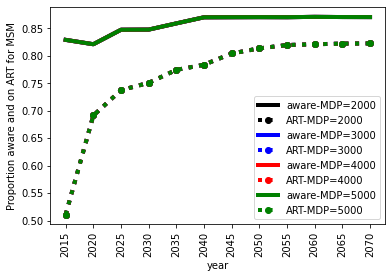


(c) (d)

**Figure A7.** HTLR Cost Function: Combination of optimal policy (testing and retention-in-care rates) for heterosexuals (a) and MSM (b), and corresponding proportion of aware and on ART for heterosexuals (c) and MSM (d) for MDP iterations of 2000 (black), 3000 (blue), 4000 (red), and 5000 (green). Results are an average of 100 runs.


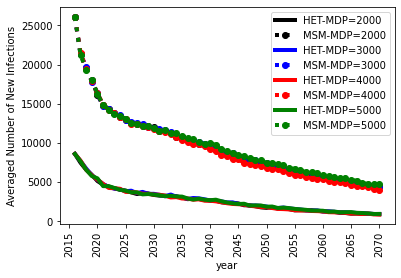

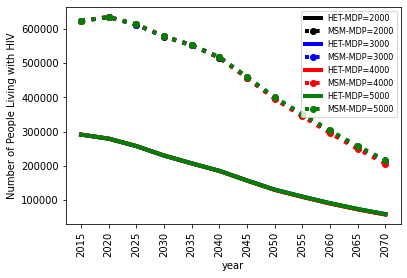


(a) (b)

**Figure A8.** HTLR Cost Function: Impact of implementing combination of optimal policy on the number of new infections (a) and the number of people living with HIV (b) for heterosexuals (solid lines) and MSM (dashed lines) for MDP iterations of 2000 (black), 3000 (blue), 4000 (red), and 5000 (green).


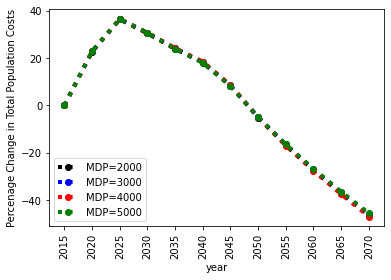


**Figure A9.** HTLR Cost Function: Percentage increment in total population cost of implementing optimal policy for MDP iterations of 2000 (black), 3000 (blue), 4000 (red), and 5000 (green). Results are an average of 100 runs. Results are an average of 100 runs.


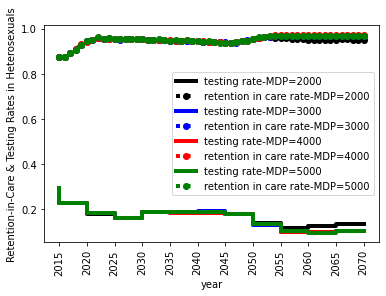

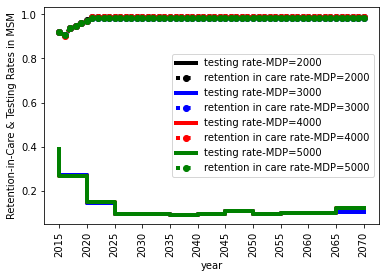


(a) (b)


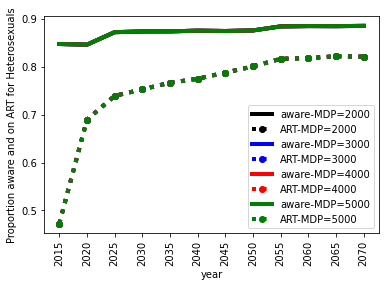

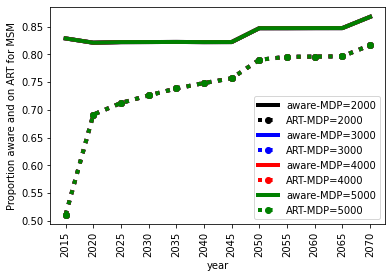


(c) (d)

**Figure A10.** LTHR Cost Function: Combination of optimal policy (testing and retention-in-care) for heterosexuals (a) and MSM (b), and corresponding proportion of aware and on ART for heterosexuals (c) and MSM (d) for MDP iterations of 2000 (black), 3000 (blue), 4000 (red), and 5000 (green). Results are an average of 100 runs.


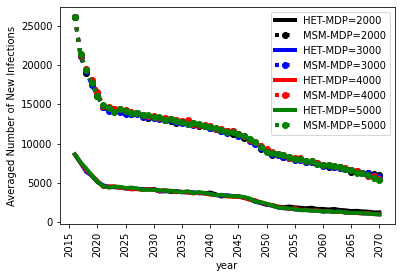

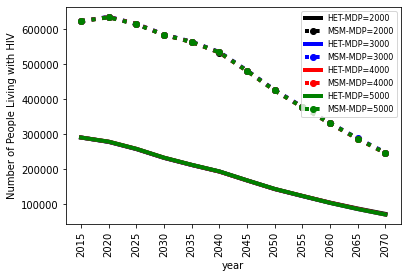


(a) (b)

**Figure A11.** LTHR Cost Function: Impact of implementing combination of optimal policy on the number of new infections (a) and the number of people living with HIV (b) for heterosexuals (solid lines) and MSM (dashed lines) for MDP iterations of 2000 (black), 3000 (blue), 4000 (red), and 5000 (green). Results are an average of 100 runs.


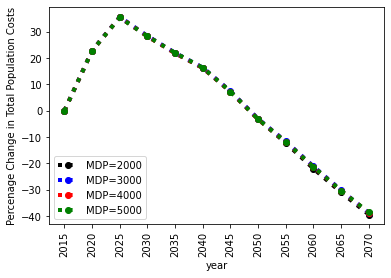


**Figure A12.** LTHR Cost Function: Percentage increment in total population cost of implementing optimal policy for MDP iterations of 2000 (black), 3000 (blue), 4000 (red), and 5000 (green). Results are an average of 100 runs.

# **Evaluating the optimality of the counter-intuitive results under the LTHR MSM scenario**

As discussed in the manuscript, comparing across the cost function assumptions, the optimal rates were generally intuitive for heterosexuals, highest testing and lowest retention-in-care in LTHR (which assumed lowest unit cost for testing and highest unit cost for retention-in-care) and lowest testing and highest retention-in-care rates in HTLR, though the differences in retention-in-care rates were modest (paper Figure 1a). However, for MSM, though the model suggested optimal rates were similar in all three cost functions, it counter-intuitively suggested a slightly lower testing rate in LTHR compared to Median and HTLR. It suggested to instead spend those resources on maintaining retention-in-care rates at the level of Median and HTLR (paper Figure 1b), such that the proportion of MSM on ART in LTHR, though lower than in Median and HTLR, was higher than the proportion of heterosexuals on ART in LTHR (paper Figure 1d). The optimality of this counter-intuitive strategy in MSM was evaluated by a counterfactual simulation run using the optimal LTHR strategy of heterosexuals for both heterosexuals and MSM (Figure A13). The number of new infections, PWH, and costs were higher in the counterfactual simulation, confirming the optimality of the policy (Figures A14 and A15). The results of this counterfactual run also suggest that the reasoning behind the counter-intuitive strategy in MSM is likely because of the higher prevalence of HIV in MSM, i.e., lowering the spending on retention-in-care would lead to more infections than lowering the spending on testing. Note also in this counterfactual run, the results for heterosexuals also change over time compared to the original (though they both start with the same optimal strategy) because of the dynamics of contact mixing between MSM and heterosexuals over time and the functionality of the model to correct for those dynamic changes and find a new optimal. A similar counterfactual run of using the MSM optimal strategy on the heterosexual risk group generated similar findings.


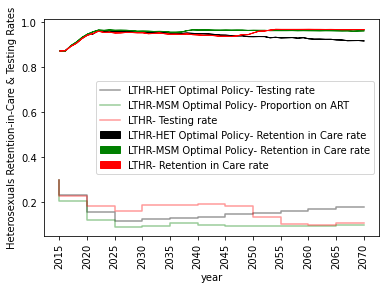

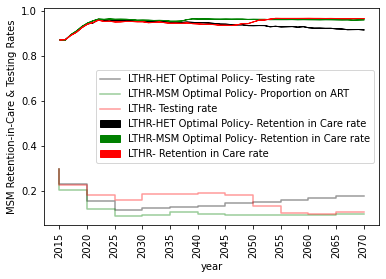


(a) (b)


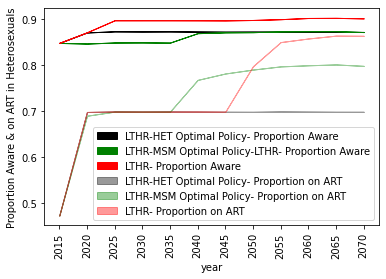

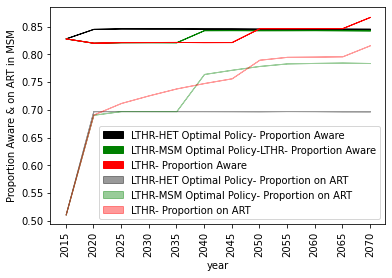


(c) (d)

**Figure A13.** Top: Optimal combination of testing rate (a) and retention-in-care rate (b). Bottom: Corresponding proportion aware (c) and proportion on ART (d).


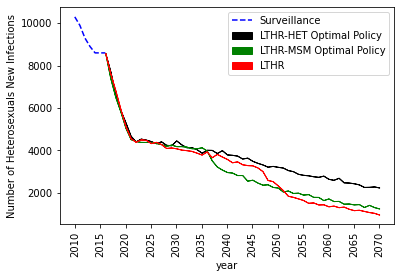

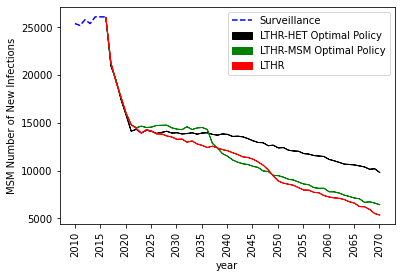


(a) (b)


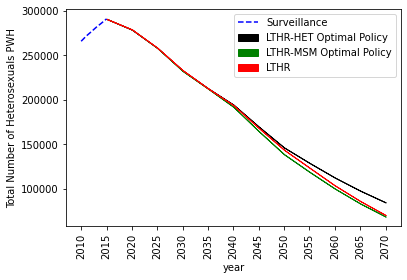

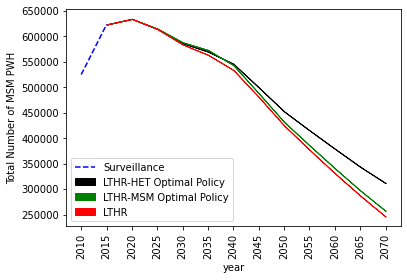


(c) (d)

**Figure A14.** Top: Number of new infections for heterosexuals (a) and MSM (b). Bottom: Number of people living with HIV for heterosexuals (a) and MSM (b).


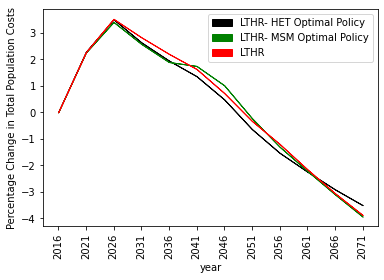


**Figure A15.** Comparing percentage change in total population cost of LTHR cost function (red) with heterosexual LTHR cost function applied to both risk groups (black) and with MSM LTHR cost function applied to both risk groups (green) all with MDP = 5000.

**Reference**

1. A. Gosavi, Simulation-based optimization: Parametric optimization techniques and reinforcement learning, (2003).
2. C. Gopalappa, P. G. Farnham, Y.-H. Chen and S. L. Sansom. Progression and Transmission of HIV/AIDS (PATH 2.0) A New, Agent-Based Model to Estimate HIV Transmissions in the United States. *Med. Decis. Making.,* **37** (2017), 224-233.
3. *Centers for Disease Control and Prevention.* Monitoring selected national HIV prevention and care objectives by using HIV surveillance data—United States and 6 US dependent areas—2010. *Citeseer*, (2012).
4. E. M. Gardner, M. P. McLees, J. F. Steiner, C. del Rio and W. J. Burman. The spectrum of engagement in HIV care and its relevance to test-and-treat strategies for prevention of HIV infection. *Clini. Infect. Dis.,* **52** (2011), 793-800.
5. R. K. Shrestha, H. A. Clark, S. L. Sansom, B. Song, H. Buckendahl, C. B. Calhoun, et al., Cost-effectiveness of finding new HIV diagnoses using rapid HIV testing in community-based organizations. *Public Health Rep.,* **123** (2008), 94-100.
6. E. U. Jacobson, K. A. Hicks, E. L. Tucker, P. G. Farnham and S. L. Sansom. Effects of reaching national goals on HIV incidence, by race and ethnicity, in the United States. *J. Public Health Manag. Pract.,* **24** (2018), E1-E8.
7. L. K. W.-D. Agero , R. D. Gorsky and G. M. Seeman. Cost of outreach for HIV prevention among drug users and youth at risk. *Drugs & Society,* **9** (1996), 185-197.
8. L. Guinness, L. Kumaranayake and K. Hanson. A cost function for HIV prevention services: is there a'u'--shape?. **5** (2007), 13.
9. L. Kumaranayake. The economics of scaling up: cost estimation for HIV/AIDS interventions. Aids, 22 (2008), S23-S33.
10. S. D. Pinkerton, L. M. Bogart, D. Howerton, S. Snyder, K. Becker and S. M. Asch. Cost of rapid HIV testing at 45 US hospitals. *AIDS patient care and STDs,* (2010)*,* 409-413.
11. P. G. Farnham, A. B. Hutchinson, S. L. Sansom and B. M. Branson. Comparing the costs of HIV screening strategies and technologies in health-care settings. *Public Health Rep.,* **123** (2008), 51-62.
12. A. Lansky, J. Christopher, O. Emeka, S. Catlainn, M. P. Joyce, E. DiNenno and N. Crepaz. Estimating the Number of Heterosexual Persons in the United States to Calculate National Rates of HIV Infection. *PloS One,* 10 (2015), e0133543.
13. A. Chandra, V. G. Billioux, C. Copen and C. Sionean. HIV risk-related behaviors in the United States household population aged 15-44 years: data from the National Survey of Family Growth, 2002 and 2006-2010. *Natl. Health Stat. Rep.,* 46 (2012), 1-19.
14. N. Khurana, E. Yaylali, P. G. Farnham, K. A. Hicks, B. T. Allaire, E. Jacobson, et al., Impact of Improved HIV Care and Treatment on PrEP Effectiveness in the United States, 2016—2020. *J. Acquir. Immune Defic. Syndr,* **78** (2018), 399-405.
15. *White House.* National HIV/AIDS strategy for the United States 2010. Available from: https://www.hivlawandpolicy.org/sites/default/files/NHAS%20strategy.pdf. Accessed April 2020.
16. A. B. Hutchinson, P. G. Farnham, S. B. Lyss, D. A. White, S. L. Sansom and B. M. Branson. Emergency department HIV screening with rapid tests: a cost comparison of alternative models. *AIDS Educ. Prev.,* **23** (2011), 58-69.
17. A. B. Hutchinson, S. F. Ethridge, L. G. Wesolowski, R. K. Shrestha, M. Pentella, B. Bennett, et al., Costs and outcomes of laboratory diagnostic algorithms for the detection of HIV. *J. Clin. Virol.,* **58** (2013), e2-e7.
18. R. K. Shrestha, L. Gardner, G. Marks, J. Craw, M. Faye, T. P. Giordano, et al., Estimating the cost of increasing retention in care for HIV-infected patients: results of the CDC/HRSA retention in care trial. *J. Acquir. Immune Defic. Syndr,* **68** (2015), 345.
19. D. Kumari, M. Chaudhary and A. K. Mishra. An Optimal Transfer of Knowledge in Reinforcement Learning through Greedy Approach. (2019).
20. T. Nieuwdorp, *Dare to Discover: The Effect of the Exploration Strategy on an Agent’s Performance,* (2017).
21. E. Even-Dar and Y. Mansour. Learning rates for Q-learning," *Journal of machine learning Research.* **5** (2003), 1-25.


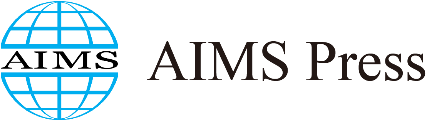
© 2021 the Author(s), licensee AIMS Press. This is an open access article distributed under the terms of the Creative Commons Attribution License (http://creativecommons.org/licenses/by/4.0)

1. This equation assumes that the outreach intervention program would be targeted towards higher-risk individuals and thus the proportion testing positive would be higher than the overall prevalence in the population, as evident by the data (presented above) in this study [5]. [↑](#footnote-ref-1)
2. The component of testing costs are taken from HOPE model technical report [6]. [↑](#footnote-ref-2)
3. Note: NAT = HIV nucleic acid amplification test [↑](#footnote-ref-3)
